# Patient Satisfaction Survey on Perioperative Anesthesia Service in University of Gondar Comprehensive Specialized Hospital, Northwest Ethiopia, 2021

**DOI:** 10.1155/2021/3379850

**Published:** 2021-10-22

**Authors:** Atsedu Endale Simegn, Debas Yaregal Melesse, Yosef Belay Bizuneh, Wudie Mekonnen Alemu

**Affiliations:** ^1^Department of Anesthesia, College of Medicine and Health Science, Wachamo University, Hosaena, Ethiopia; ^2^Department of Anesthesia, College of Medicine and Health Science, University of Gondar, Gondar, Ethiopia

## Abstract

**Background:**

Patient satisfaction is a crucial component in determining the quality of healthcare in anesthesia services. This study aimed to assess patient satisfaction and associated factors on perioperative anesthesia services.

**Methods:**

Institutional-based cross-sectional study was conducted from May 16 to June 22, 2021, at the University of Gondar Comprehensive Specialized Hospital. Data were entered into Epi-data version 3.1 and exported to STATA version 14.1. The strength of the association was presented using an adjusted odds ratio with a 95% confidence interval, and a *p* value <0.05 was considered as statistically significant.

**Results:**

A total of 398 patients were included in this study with a response rate of 98%. The overall proportion of patients who were satisfied with perioperative anesthesia service was 74% (95% CI: 69–78). Patients who received regional anesthesia were 2.8 times satisfied than those who received general anesthesia (AOR = 2.8, 95% CI: 1.42–5.36). Patients who obtained adequate information was 3.14 times (AOR = 3.14, 95% CI: 1.71–5.74) satisfied than that of the counterpart. Adults who did not feel pain during induction of anesthesia were 2.7 times (AOR = 2.7, 95% CI: 1.43–5.08) satisfied than an adult who felt pain during induction of anesthesia. *Conclusion and Recommendations*. The overall patients' satisfaction on perioperative anesthesia service was 74%. Patients who underwent operation with regional anesthesia, obtained adequate information about anesthesia, visited by anesthetists after operations, did not have nausea/vomiting, did not feel pain during induction, and patients who did not feel pain immediately after operation were satisfied than the counterparts. We recommended that the anesthetists must give attention to reduce the factors that decrease the satisfaction level of the surgical patients.

## 1. Background

Patient satisfaction refers to how satisfied patients are with the services delivered in terms of meeting their requirements and expectations. It is described as a subjective assessment of the service obtained considering the individual's expectations and a crucial component in determining the quality of healthcare. Patient satisfaction may have a significant impact on a variety of areas of their behavior, including overall healthcare resource consumption, adherence to treatments, and the consistency of their connection with practitioners [1–3]. Several forms of anesthesia, including regional anesthesia, general anesthesia, and a possible combination of regional and general anesthesia, are widely used throughout modern surgery. Therefore, comprehensive evaluation of patient satisfaction after anesthesia services is an important parameter for quality control and continuous improvement in-hospital care [4, 5]. Patient satisfaction provides patient-centered care in a culture that accepts people for whom they are and where they are in their life cycle by satisfying their needs at that time, in line with the health system's objective of caring for patients' bodies, minds, and spirits and also to have a good impact on personnel, the community, and the organization's health [6]. The majority of studies showed that the magnitude of patient satisfaction on anesthesia service was high. Some studies showed that the overall number of patients who were happy with anesthesia services was low. Therefore, it is the responsibility of every member of staff to provide the best possible care, and many healthcare organizations should consider measuring patient satisfaction to be an important part of quality assessment [7–9]. Patient dissatisfaction can harm healthcare providers and healthcare facilities [10]. For every unhappy patient who express his or her dissatisfaction to your team, many more patients will keep silent and most likely never return to your practice, and others will share their negative experiences with their friends and family [11, 12]. Different studies showed that the following factors were associated with patient satisfaction in anesthesia services; age, educational level, and marital status, as well as expectations; the information provided emotional support, anesthesia duration, physical discomfort, perioperative complications, postoperative pain, anesthetic care during the perioperative period, postoperative visits, and contact with the patient can all have an impact on patient satisfaction [11, 13–17]. Patients with ASA I-II were significantly less satisfied than ASA III patients. Postoperative visiting of surgical patients with anesthetists and delivering adequate information improve patient satisfaction [18–21]. In Ethiopia, there were studies conducted on patient satisfaction on anesthesia services and showed that the satisfaction level was low [16, 22–25]. Therefore, this research also intended to assess patient satisfaction and associated factors on perioperative anesthesia service in the set up in 2021, according to the recommendations of the previous studies.

## 2. Methods

### 2.1. Study Setting

The study was conducted at the University of Gondar Comprehensive Specialized Hospital (UoGCSH) in recovery rooms and the surgical wards. Gondar is one of the Ethiopian ancient towns, which is the capital city of the Central Gondar administrative zone in Amhara regional state, located 738 km northwest of Addis Ababa and 180 km from Bahir Dar which is the capital city of Amhara regional state. The hospital is a comprehensive, specialized, and teaching hospital which is found in Gondar town and provides health services for more than seven million people in the catchment area. The hospital has 630 beds, 7 major operation rooms, two obstetric operation rooms, and a fistula unit for the gynecological procedures which contain two operation rooms, an ophthalmic unit which has one operating room, and this hospital also has four intensive care units. According to the UoGCSH planning and program coordination office report, 4,125 patients were operated on under anesthesia in 2020.

### 2.2. Study Design and Period

An institutional-based cross-sectional study was conducted from May 16 to June 22, 2021.

### 2.3. Sample Size and Sampling Procedure

The sample size was calculated using a single population proportion formula based on a previous study performed in Hawassa University Comprehensive and Specialized Hospital (HUCSH), Ethiopia, which showed that the magnitude of satisfaction was 60% [23]. Assume a 95% of confidence interval with a 5% margin of error with a nonresponse rate of 10%, and finally, the sample size for the study was calculated as

(1) $$\begin{array}{c} n=\frac{{\left(Z\alpha /2\right)}^{2}\times p\left(1-p\right)}{d^{2}},\\ n=\frac{{\left(1.96\right)}^{2}\times \left(0.6\left(\;1-0.6\right)\right)}{0.05^{2}}, \end{array}$$

where *n* is the required sample size; *p*=60% is the magnitude of satisfaction from HUCSH;^,^*Zα*/2 is the value (*Z*-statistic) at the 95% confidence level (*α* = 0.05) which is 1.96; *d* is the margin of error 5% (0.05) with 10% nonresponse rate; and the final sample size was *n* = 406. All adult emergency and elective patients who were operated on under anesthesia (trauma and orthopedics, obstetric and gynecologic, ear, nose, and throat (ENT), maxillofacial, and general surgeries) were included till the calculated sample size was reached.

### 2.4. Data Collection Procedure and Quality Assurance

A pretested semistructured questionnaire was used for data collection. A questionnaire used to assess satisfaction was adapted from the Leiden Perioperative Care Patient Satisfaction Questionnaire which is a valid and reliable perioperative patient satisfaction assessment tool that has three dimensions (staff-patient relationship, information provision, and fear and concern). In this study, these 3 dimensions were assessed by using a 5-point Likert scale which were expressed as follows:1, strongly dissatisfied; 2, dissatisfied; 3, neutral; 4, satisfied; and 5, strongly satisfied [7]. This scale was dichotomized as satisfied and dissatisfied based on the demarcation threshold formula. The questionnaires were first prepared the English language and translated into Amharic by language experts. The data were collected by two BSc anesthetists using chart review and face-to-face interviews. Regular supervision and follow-up were done during data collection. The data completeness and consistency were checked by the principal investigator daily.

### 2.5. Variables of the Study

The dependent variable of this study was the level of patient satisfaction, which was recorded by a five-point Likert scale. Patients were satisfied with perioperative anesthesia services who scored above the cut point based on the demarcation threshold formula ((total highest score − total lowest score)/2) + (total lowest score) [26–31]. The independent variables were sociodemographic (age, sex, and educational status), surgical and anesthetic-related factors (types of anesthesia, types of surgery, and ASA status), preoperative anesthesia-related factors (self-introduction of anesthetist, give chance for the patient to ask a question, give adequate information about the type of anesthesia, and give chance for the patient to choose the type of anesthesia), intraoperative anesthesia-related factors and reception in the operation room (reception, pain during induction, privacy, awareness, and pain during the operation and immediately after operation), postoperative anesthesia-related factors, and postoperative anesthetist visit (pain during swallowing food, hoarseness of voice, depression, nausea, vomiting, shivering, postoperative anesthesia visit, number of visit, and treat complain during visit).

### 2.6. Data Processing and Analysis

After checking the data for completeness and consistency, the data were coded and entered using Epi-data version 3.1 and transfered to STATA version 14.1 software for analysis. Normality was checked using the Shapiro–Wilk test. Multicollinearity was also checked using the variance inflation factor and found that there was no multicollinearity since all variables had variance inflation factor <5 and tolerance less than 10%. Then, the frequency, percentage, and cross-tabulation with different variables were performed. Variables of interest were fitted using the logit commands in STATA version 14.1, and model fitness was checked by the Hosmer and Lemeshow goodness of fit test. Finally, the contributing factors were analyzed using bivariate binary logistic regression and multivariate binary logistic regression. Variables with a *p* value <0.25 were fitted in a multivariate binary logistic regression, and a *p* value less than 0.05 was taken as statistically significant factor for satisfaction.

## 3. Results

### 3.1. Sociodemographic and Clinical Characteristics of Study Participants

A total of 398 adult patients have participated in this study with a response rate of 98%. Nearly 2/3 of the study participants, 260 (65.33%), were females. Around half (51.76%) of the study participants were range within the age of 18–29 years old. Regarding ethe ducational level, 33.42% had no formal education. A majority (83.42%) of the study participants underwent major surgery. Among 398 patients, 62.81% underwent regional anesthesia (Table 1).

### 3.2. Intraoperative Anesthesia-Related Factors and Reception in the Operating Theatre

From the total of respondents, 85.43% clarified that the reception of anesthetist was good, and 89.7% of study participants felt that privacy was kept. Most of the study participants did not feel pain during the operation and immediately after operation, 92.21% and 81.41%, respectively (Table 2).

### 3.3. Overall Patient Satisfaction with Perioperative Anesthesia Services

**Figure 1:**
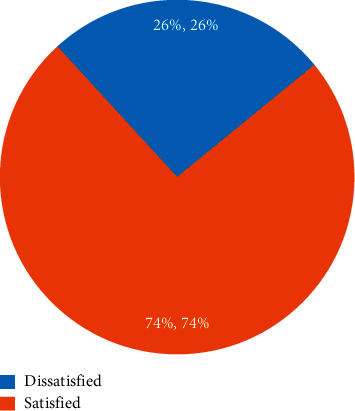
Overall patient satisfaction of anesthesia services at University of Gondar Comprehensive Specialized Hospital, northwest Ethiopia, 2021 (*N* = 398).

The overall satisfaction level of patients with perioperative anesthesia care was 74% (95% CI: 69–78) (Figure 1).

### 3.4. Factors Associated with Patient Satisfaction on Perioperative Anesthesia Services

In multivariate binary logistic regression analysis, factors such as types of anesthesia, give adequate information about anesthesia, absence of pain during induction, absence of pain immediately after the operation, absence of nausea and vomiting, and anesthetist visit after operation were found to be significantly associated with perioperative anesthesia service patient satisfaction. The odds of having satisfaction for those adults who received regional anesthesia were 2.8 times higher than that of those adults who received general anesthesia (AOR = 2.8, 95% CI:1.42–5.36). The odds of having satisfaction for adults who obtained adequate information were 3.14 times (AOR = 3.14, 95% CI: 1.71–5.74) higher than that of the counterpart. The odds of having satisfaction for adults who did not feel pain during induction of anesthesia was 2.7 times (AOR = 2.7, 95% CI: 1.43–5.08) higher than adults who felt pain during induction of anesthesia. The odds of having anesthesia service satisfaction for those who did not feel pain immediately after the operation was 2.9 times (AOR = 2.9, 95% CI: 1.47–5.74) more likely satisfied than those who felt pain immediately after operation. In addition, nausea/vomiting and anesthetist visits after operation were significant predictors of patient's satisfaction in anesthesia service. Patients who had no nausea/vomiting after operation had 2.3 times higher odds of having satisfaction on perioperative anesthesia service compared to the counterpart (AOR = 2.3, 95% CI: 1.25–4.17). The odds of having satisfaction for those who were visited by anesthetist after operation were 5.5 times more likely satisfied than those who were not visited by an anesthetist (AOR = 5.5, 95% CI: 2.89–10.52) (Table 3).

## 4. Discussion

In this study, the overall satisfaction of patients on perioperative anesthesia service was 74% (95% CI: 69–78). The finding was similar to a study performed in India [32]. This similarity might be due to a similar study design and lower socioeconomic status. Our study finding was higher compared with studies performed in Hawassa [23] and Eritrea [7]. This difference might be due to the large sample size, standard perioperative management of patients to decrease patient compliant, and low compared to previous findings in Ethiopia, Gondar [16] and Mekelle [22]. This discrepancy might be due to the large sample size, use of a different questionnaire which is the Leiden Perioperative Care Patient Satisfaction Questionnaire, and in addition low compared with Greek [33] and Nigeria [34]. The difference might be attributed due to socioeconomic deference, organizational, structural infrastructure, and capacity differences between the countries.

This study identified that there were different independent variables associated with anesthesia service satisfaction in the University of Gondar Compressive Specialized Hospital.

In our study, the patient who received regional anesthesia was more likely satisfied compared to patient who received general anesthesia type. This finding was consistent with previous studies [35]. This might be due to the reason that the possibility of speaking with relatives during the immediate postoperative period, staying awake, the absence of pain during the immediate postoperative period, ability to eat, and drink early after anesthesia and ability to phone early after anesthesia in addition to general anesthesia involves a complete loss of consciousness, while regional anesthesia numbs a specific area of the body without altering brain or breathing functions.

Patients who gain adequate information were more likely satisfied than a patient who had not gained adequate information about anesthesia. This finding was similar to previous studies [16, 23]. The possible explanation might be giving enough information about the side effect, and advantages of anesthesia may give psychological preparation for surgery; this may alleviate anxiety and postoperative complications which in turn reduce the length of hospital stay.

Pain during induction of anesthesia negatively associated with patient satisfaction with anesthesia services. This finding was supported by previous findings [16, 22]. It might be due to the administration of intravenous anesthetic agents like propofol during GA and pain during needle insertion, while giving regional anesthesia might dissatisfy patients.

The patients who felt pain immediately after operation were less likely satisfied than those who did not feel pain immediately after operation. This is in line with previous studies [26, 36, 37]. The reason might be the fact that pain interferes with falling asleep, staying asleep, and doing activities in bed. Furthermore, it affects mood and emotions, and patients be anxious, depressed, frightened, and helpless. It has also side effects such as nausea, drowsiness, and dizziness.

Patients who had postoperative nausea and vomiting were less likely satisfied than the counterpart, supported with the previous study [23]. This might be due to its interference with recovery such as eating and mobilizing and leads to potentially delaying recovery. It also has functional interference with sleep, appetite, physical activity, and general interactions.

Patients who had postoperative visits were more satisfied than those who did not get visit. This finding was similar to a study performed in Switzerland [20]. This could be because of the encouragement of patients, and patients might get dealing with their criticism at the right time.

### 4.1. Strength and Limitation of the Study

The study used a relatively large sample size and high response rate than the previous studies, many of the previous findings in Ethiopia were only descriptive, and we used regression analysis for associated factors were the strengths of our study. Due to cross-sectional nature of the design, because the exposure and outcome are simultaneously assessed, there is generally no evidence of a temporal relationship between exposure and outcome. The limitation of this study was that we did not include patients who were discharged before 24 hrs of operation.

### 4.2. Conclusion and Recommendations

The overall patients' satisfaction on perioperative anesthesia service was 74%. Patients who underwent operation with regional anesthesia, obtained adequate information about anesthesia, visited by anesthetists after operations, did not have nausea/vomiting, did not feel pain during induction, and patients who did not feel pain immediately after operation were satisfied than the counterparts. We recommended that the anesthetists must give attention to reduce the factors that decrease the satisfaction level of the surgical patients.

## Figures and Tables

**Table 1:** Sociodemographic and clinical characteristics of participants at University of Gondar Comprehensive Specialized Hospital, northwest Ethiopia, 202l (*N* = 398).

Variables		Level of satisfaction		
		Frequency (*N*)	Satisfied (%)	Dissatisfied (%)
Sex	Male	138 (34.67)	101 (73.2)	37 (26.8)
	Female	260 (65.33)	192 (73.8)	68 (26.2)

Age	18–29	206 (51.76)	149 (72.3)	57 (27.7)
	30–49	139 (34.92)	103 (74.1)	36 (25.9)
	50–65	42 (10.55)	33 (78.5)	9 (21.5)
	>65	11 (2.76)	8 (72.8)	3 (27.2)

Educational status	No formal education	133 (33.42)	102 (76.7)	31 (23.3)
	Primary	105 (26.38)	71 (67.6)	34 (32.4)
	Secondary	94 (23.62)	71 (75.5)	23 (24.5)
	Higher education	66 (16.82)	49 (74.2)	17 (25.8)

Types of anesthesia	General	148 (37.19)	97 (65.5)	51 (34.5)
	Regional	250 (62.81)	196 (78.4)	54 (21.6)

Types of surgery	Minor	66 (16.58)	52 (78.8)	14 (21.2)
	Major	332 (83.42)	241 (72.6)	91 (27.4)

ASA status	ASAI	212 (53.27)	158 (74.5)	54 (25.5)
	ASAII	166 (41.71)	123 (74.0)	43 (26)
	ASAIII	20 (5.03)	12 (60)	8 (40)

Introduce him or herself	Yes	195 (48.99)	157 (80.5)	38 (19.5)
	No	203 (51.01)	136 (67.0)	67 (33.0)

Give adequate information about	Yes	252 (63.32)	213 (84.5)	39 (15.5)
	No	146 (36.68)	80 (55.0)	66 (45.2)

Anesthesia chance to choose anesthesia	Yes	47 (11.81)	39 (83.0)	8 (17.0)
	No	351 (88.19)	254 (72.4)	97 (27.6)

Chance to ask question	Yes	105 (26.38)	89 (84.8)	16 (15.2)
	No	293 (73.62)	204 (69.7)	89 (30.4)

ASA, American Society of Anesthesiologists.

**Table 2:** Intraoperative anesthesia-related factors and reception in the operating theatre at the University of Gondar Comprehensive Specialized Hospital, northwest Ethiopia, 2021 (*N* = 398).

Variables		Level of satisfaction		
		Frequency, *N* (%)	Satisfied (%)	Dissatisfied (%)
Reception of anesthetist in the OR	Good	340 (85.43)	255 (75.0)	85 (25.0)
	Bad	58 (14.57)	38 (65.5)	20 (34.5)

Pain during induction of anesthesia	Yes	136 (34.17)	93 (68.4)	43 (31.6)
	No	262 (65.83)	200 (76.3)	62 (23.7)

Anesthetists consider your privacy in the operation room	Yes	357 (89.70)	266 (74.5)	91 (25.5)
	No	41 (10.30)	27 (65.9)	14 (34.1)

Feel pain during operation	Yes	31 (7.79)	22 (71.0)	9 (29.0)
	No	367 (92.21)	271 (74.0)	96 (26.1)

Pain immediately after operation	Yes	74 (18.59)	35 (47.3)	39 (52.7)
	No	324 (81.41)	258 (79.6)	66 (20.3)

OR, operation room; *N*, number.

**Table 3:** Bivariate and multivariate binary logistic regression analyses of contributing factors in the University of Gondar Comprehensive Specialized Hospital, northwest Ethiopia, 2021 (*N* = 398).

Variables	Categories	COR (95% CI)	AOR (95% CI)
Types of anesthesia	General	1.00	1.00
	Regional	1.9 (1.21, 3.00)	2.8 (1.41, 5.22)^*∗∗*^

Introduce him/her to patient	Yes	1.00	1.00
	No	0.49 (0.31, 0.77)	0.59 (0.31, 1.09)

Give adequate information	Yes	1.00	1.00
	No	0.22 (0.13, 0.35)	0.31 (0.17, 0.58)^*∗∗*^

Chance to ask question	Yes	1.00	1.00
	No	0.41 (0.22, 0.74)	0.80 (0.38, 1.71)

Reception of anesthetist in the OR	Good	1.00	1.00
	Bad	0.63 (0.34, 1.14)	0.59 (0.28, 1.21)

Pain during induction	Yes	1.00	1.00
	No	1.49 (0.94, 2.36)	2.70 (1.43, 5.08)^*∗∗*^

Considering privacy	Yes	1.00	1.00
	No	0.65 (0.33, 1.31)	0.60 (0.26, 1.37)

Pain immediately after surgery	Yes	1.00	1.00
	No	4.4 (2.56, 7.40)	2.9 (1.47, 5.74)^*∗∗*^

Nausea and vomiting	Yes	1.00	1.00
	No	2.73 (1.70, 4.39)	2.3 (1.25, 4.17)^*∗∗*^

Anesthetist visit after surgery	No	1.00	1.00
	Yes	4.35 (2.56, 7.40)	5.5 (2.89, 10.52)^*∗∗*^

l.00, reference; OR, operation room; COR, crude odds ratio; AOR, adjusted odds ratio; CI, confidence interval. ^*∗*^*p* value <0.05, ^*∗∗*^*p* value <0.01.

## Data Availability

The data generated or analyzed during this study are included within this article.

## Abbreviations

PONV: Postoperative nausea and vomiting
Hr.: Hour
GA: General anesthesia
COR: Crude odds ratio
AOR: Adjusted odds ratio
CI: Confidence interval
ASA: American Society of Anesthesiologists.
